# Gender differences and lung cancer risk in occupational chefs: analyzing more than 350,000 chefs in Taiwan, 1984–2011

**DOI:** 10.1007/s00420-018-1358-8

**Published:** 2018-09-18

**Authors:** Pei-Chen Lin, Chiung-Yu Peng, Chih-Hong Pan, Pi-I Debby Lin, Ming-Tsang Wu

**Affiliations:** 1 Department of Public Health, College of Health Sciences, Kaohsiung Medical University, Kaohsiung, Taiwan; 2 Research Center for Cijin Cohort, Kaohsiung Medical University, Kaohsiung, Taiwan; 3 Research Center for Environmental Medicine, Kaohsiung Medical University, Kaohsiung, Taiwan; 4 Institute of Labor, Occupational Safety and Health, Ministry of Labor, Taipei, Taiwan; 5 Harvard TH Chan School of Public Health, Boston, MA USA; 6 Department of Family Medicine, Kaohsiung Medical University Hospital, Kaohsiung Medical University, Kaohsiung, Taiwan; 7 Graduate Institute of Clinical Medicine, College of Medicine, Kaohsiung Medical University, Kaohsiung, Taiwan

**Keywords:** Lung cancer, Occupational chefs, Gender, Cooking oil fumes

## Abstract

**Objectives:**

Cooking oil fumes (COFs) contain many carcinogens. We investigated the association between COFs and incidence risk of any cancer and lung cancer in chefs.

**Methods:**

We identified Chinese food chefs and non-Chinese food chefs from Taiwan’s national database of certified chefs in 1984–2007. Of them, 379,275 had not been diagnosed as having any cancer before chef certification. We followed them in Taiwan’s Cancer Registry Database (1979–2010) and Taiwan’s National Death Statistics Database (1985–2011) for any newly diagnosed cancer or lung cancer.

**Results:**

378,126 and 379,215 chefs were included for risk analysis of cancer and lung cancer, respectively. 6099 chefs developed cancer and 339 developed lung cancer over the follow-up periods of 4,183,550 and 4,220,163 person-years, respectively. Compared to non-Chinese food chefs, the adjusted IRR of cancer for Chinese food chefs was 1.69 (95% CI 1.51–1.89). For lung cancer, the risk was significantly higher among Chinese food chefs who had been certified for more than 5 years (adjusted IRR 2.12, 95% CI 1.32–3.40). This increased risk was pronounced in female chefs (adjusted IRR 4.73, 95% CI 1.74–12.86).

**Conclusions:**

Chinese food chefs had an increased risk of cancer and lung cancer, particularly in females.

**Electronic supplementary material:**

The online version of this article (10.1007/s00420-018-1358-8) contains supplementary material, which is available to authorized users.

## Introduction

Lung cancer, the leading cause of cancer death worldwide, caused 1.6 million deaths in 2012 (IARC 2014). This disease once occurred primarily in men, who traditionally have had a high prevalence of cigarette smoking, though the gender gap has been narrowing since the 1980s (Sun et al. 2007). While the incidence of lung cancer has been leveling off among men, women have seen a significant upward trend in its incidence (Chen et al. 2016; Egleston et al. 2009).

The two most common histological subtypes of lung cancer are lung squamous cell carcinoma (LSCC) and lung adenocarcinoma (LA). Although all subtypes of lung cancer have been linked to cigarette smoking, the linkage between cigarette smoking and LSCC is stronger than it is for LA (Sun et al. 2007). This discrepancy suggests possible involvement of other important risk factors from the environment in the development of LA in never smokers.

In 2007, Sun et al. (2007), citing global cancer statistics data, estimated that ~ 15% of the cases of lung cancer in men and 53% in women worldwide, could not be attributed to cigarette smoking. That figure makes up ~ 25% of all lung cancer cases globally (Parkin et al. 2005; Sun et al. 2007). According to Taiwan’s Adult Smoking Behavior Surveillance System (ASBS), the smoking rate among men ranges in Taiwan 21–39% and in women between 1.5 and 6.7%, respectively (HPA 2014). This wide discrepancy in smoking rates between Taiwanese men and women may also influence the attributable rate of cigarette smoking on lung cancer risk (Lo et al. 2013).

The etiology of lung cancer in never smokers is complex. Besides the risk factor of second-hand smoke (Lo et al. 2013; Sun et al. 2007), some epidemiological studies have linked exposure to cooking oil fumes (COFs) to lung cancer in nonsmoking women in China, Taiwan, Hong Kong, and Singapore (Ko et al. 2000; Lee and Gany 2013; Mu et al. 2013; Sun et al. 2007). An updated meta-analysis of population-based and hospital-based case–control studies showed an odds ratio of 1.74 (95% CI 1.57–1.94) for lung cancer among nonsmoking Chinese women exposed to COF compared to those not exposed to COFs (Xue et al. 2016).

Chinese cooking predominately involves stir frying, pan frying (sautéing) and deep frying in oil at high temperatures, which generates COFs (Lee et al. 2000). COFs contain many chemicals, including polycyclic aromatic hydrocarbons (PAHs) such as benzo[α]pyrene and aldehydes such as *t,t*-2,4-decadienal (*t,t*-2,4-DDE), which are known or suspected carcinogens (Fullana et al. 2004a, b; Guillen et al. 2005). Although many epidemiological studies suggest exposure to COFs is associated with lung cancer risk among nonsmoking women, the International Agency of Research on Cancer (IARC) still categorizes emissions from high-temperature frying as Group 2A carcinogens (probable carcinogenic), indicating limited evidence of carcinogenicity in humans (IARC 2010). Thus, we conducted a nationwide retrospective epidemiological study in occupational chefs, potentially at highest risk of exposure to COFs, to investigate the effect of COFs on incidence of cancer and lung cancer in both males and female chefs.

## Materials and methods

### Database of certified cook registration

This study linked certification dates and employment data of chefs listed in Taiwan’s Ministry of Labor’s national database of certificated chefs to two national health status-related databases belonging to Taiwan’s Ministry of Health and Welfare to calculate the risk of any cancer and lung cancer in Chinese food chefs and non-Chinese food chefs.

Following STROBE guidelines for observational studies (Supplementary Materials eTable 1) (von Elm et al. 2007), we first identified professional chefs from a database belonging to Taiwan’s Ministry of Labor, which provides professional certificates for chefs in Taiwan. Collection of the data began in paper form in 1972 and was continued in digital form in 1984. This study used the digital database from 01 January 1984 to 31 December 2007. The demographic data collected from this database were encrypted ID number, gender, birthday, date of professional certification (year/month/day), and category of certified skill. Certified skills were categorized into Chinese food cooking (exposure group), typically involving frying in hot oils such as deep frying and stir frying, and non-Chinese cooking (non-exposure group). The non-Chinese cooking group consisted of chefs working within wide range of job categories related to different kinds of food preparation and processing before preparing Chinese cuisines or other non-Chinese cuisines. Those job categories were subdivided into seven job activities, including rice processing, aquatic product processing, processing by baking, western-style baking, wheat flour processing, meat processing, and catering services. Unlike Chinese cooking, these categories of cooking are less likely to involve exposure to COFs. In addition, both Chinese and non-Chinese food chefs and food workers should pass different professional skills and food hygiene knowledge tests in written and in practice to receive to different certificates from the Taiwan government. Because both Chinese and non-Chinese food chefs are considered workers, they are required to adhere the labor law implemented by Taiwan’s Ministry of Labor to work 8 h a day for 6 days and 1–2 days off in a period of 1 week.

**Fig. 1:**
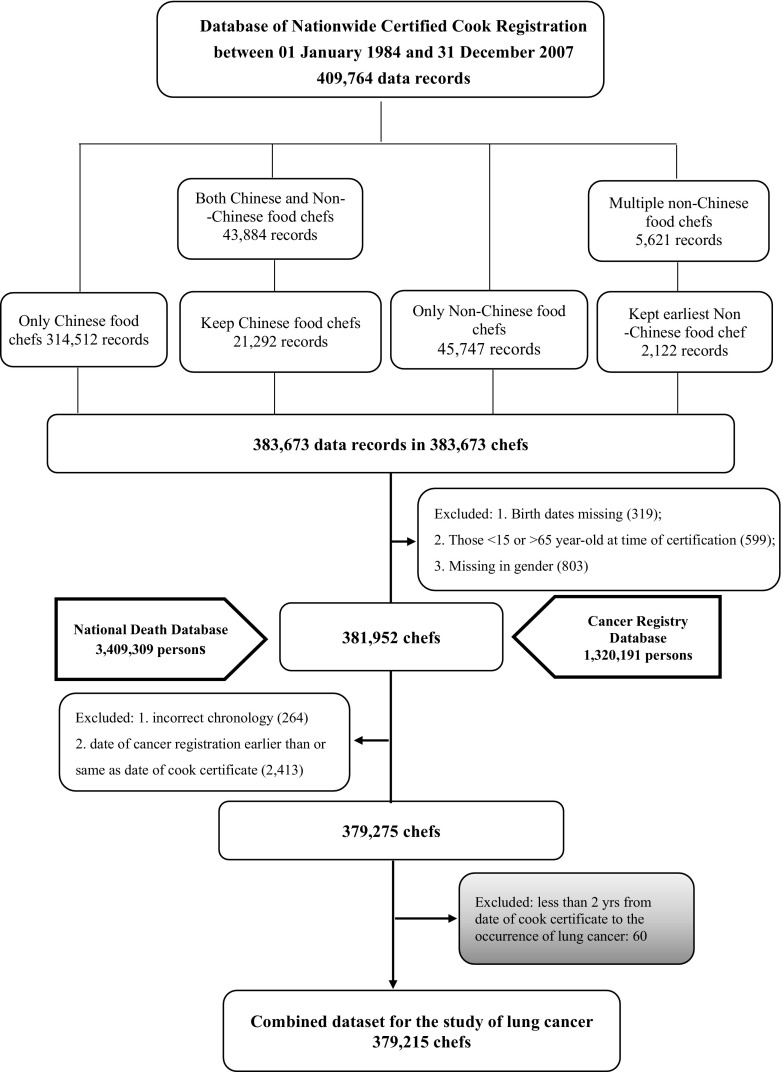
The follow-up chart of lung cancer sample selection

In total, 409,764 records were found in the certified cook registration database. The dataset had to be cleaned before being linked to the two health status datasets, because some subjects had received two different certificates at different times (Fig. 1). If a subject received certificates in Chinese and non-Chinese food cooking in different years, he or she was assigned to the Chinese food chef group (exposure group). If a subject had more than one type of non-Chinese cooking certificate, he or she was assigned to the category representing the first certificate he or she received and placed in the non-Chinese cooking group (non-exposure group). After assigning the chefs into one category only, we were left with 383,673 records. We further excluded subjects with missing birthdates or certification dates (*n* = 319), subjects who obtained certificates > 65 or < 15 years old (*n* = 599), and subjects whose gender status was missing (*n* = 803). This further exclusion left 381,952 records to be linked to the two national health status-related databases (Fig. 1).

### Linkage to Taiwan’s cancer registry file and cause-of-death file

After cleaning the nationwide certified chef database, we linked its data via encrypted ID numbers to Taiwan’s Cancer Registry Database and Taiwan’s National Death Statistics Database, both maintained by Taiwan’s Ministry of Health and Welfare. The two national vital statistics datasets have been reported to be reliable and comprehensive by several epidemiological studies (Lai et al. 2010; Yang et al. 2002). In 1979, all hospitals nationwide with in-patients beds ≥ 50 were required to start reporting all diagnosed malignancies to the Ministry of Health and Welfare. These are recorded in the Taiwan’s Cancer Registry Database (Chiang et al. 2015). We linked chef data to Taiwan’s Cancer Registry Database, containing items of encrypted ID number, gender, birthdate, date of cancer diagnosis, ICD-9-CM code and pathological histological code, collected from 01 January 1979 to 31 December 2010. After excluding data with missing encrypted ID numbers and those with two different cancers registered at different dates, we were left with 1,320,191 files (Fig. 1). Their data was also linked to the National Death Statistics Database, containing encrypted ID number, gender, birthdate, expiry date and cause of death (ICD-9 or ICD-10 code). We applied to the Ministry to use data collected from 01 January 1985 to 31 December 2011. After excluding data with missing encrypted ID numbers and unclear dates of birth or death, we were left with 3,409,309 records of death.

The three national databases were linked using encrypted ID numbers starting from the certified cook database. After further excluding those with unclear dates of cancer diagnosis, death, and certification (*n* = 264) and those whose cancers were diagnosed before obtaining chef certificates (*n* = 2413), we were left with 379,275 subjects. To test the risk of cancer and lung cancer only, we chose the eligible subjects in the final analysis separately (Fig. 1 and eFigure 1). For cancer risk, we further excluded any subject who developed cancer (*n* = 1149) within 2 years of receiving a chef certificate taking into account possible latent development of disease leaving us with 378,126 chefs to be included in our study of cancer risk (eFigure 1). For lung cancer risk, we excluded lung cancer only (*n* = 60) within 2 years of receiving a chef certificate taking into account the latent period leaving use with 379,215 chefs to be included in our study of lung cancer risk (Fig. 1). The protocol for this study was approved by the Institutional Review Board (IRB) of Kaohsiung Medical University Hospital. Because the subject identifiers in the merged dataset were scrambled before analysis to protect confidentiality, the requirement for written or verbal consent from subjects was waived.

### Statistical analysis

We estimated incidence rate (IR) of cancer and lung cancer, defined as the occurrence of an event (cancer or lung cancer) over person-time, in Chinese food chefs (exposure group) and non-Chinese food chefs (non-exposure group). Person-time for any cancer risk as the denominator was calculated from date of certification to the date of diagnosis of any cancer (ICD-9-CM: 140–165, 170–171,173–175, 179–195, 199, and 200–208), death, or the end of the study (31 December 2011), whichever came first, and the numerator was the count of diagnoses of cancer. For lung cancer risk, person-time as the denominator was calculated from date of certification to the date of diagnosis of lung cancer (ICD-9-CM: 162), any other cancer, death, or the end of the study (31 December 2011), whichever came first, and the numerator was the count of diagnoses of lung cancer. The incidence rate ratio (IRR) of cancer and lung cancer was calculated to compare the rate ratio difference between the exposure group and non-exposure group for cancer and lung cancer, respectively, using the Poisson regression method before and after adjusting for other covariates, including gender and age range (15–39, 40–59 and ≥ 60 years old). For age, we categorized the subject into three age groups, because, in Taiwan, individuals can be qualified as occupational chefs, after the age of 15 years old and most of them receive their licenses before 40 years old.

For lung cancer, the exposure group was subdivided into those with certificates for less than and equal to 5 years and ones with certificates for more than 5 years. Using non-Chinese food chefs (non-exposure group) as the comparison group, we calculated IRR of lung cancer for different exposure groups. In addition to calculating IRRs dichotomized by gender, we also calculated IRRs by subtypes of lung cancer. We divided the histological types of lung cancer into three main categories using pathological histological codes: LA, LSCC, and “Others” (eTable 2). Because the case numbers of LSCC and “Others” were smaller than LA, we combined these cases together into a non-LA category for further analyses (Sun et al. 2007). In the sensitivity analysis, LSCC patients were also analyzed alone. All data were stored and analyzed in the Health and Welfare Data Science Center, Ministry of Health and Welfare (HWDC, MOHW), Taiwan (case number: H103101). All statistical operations were performed using SAS 9.4 inside Health and Welfare Data Science Center governed by Taiwan’s Ministry of Health and Welfare. All *P* values were two-sided and considered significant if < 0.05.

## Results

A total of 378,126 and 379,215 chefs were included in our analysis of any cancer risk and lung cancer risk, respectively (Fig. 1). Of these chefs, 330,947 and 331,981 were Chinese food chefs, respectively, accounting for 87.5% of the entire population. 47,179 and 47,234 non-Chinese food chefs were included in our analysis of cancer and lung cancer risk groups, respectively (Table 1). Most of the chefs (68.4%) in this study were women, particularly Chinese food chefs (69.9%). Most (72.7%) had obtained certificates and started cooking between 15 and 39 years old. Chinese food chefs were older than non-Chinese food chefs at the beginning of their careers (32.13 vs. 23.28 years old) (Table 1).

**Table 1:** Characteristics of occupational chefs in the studies of cancer risk and lung cancer risk

Cancer risk	Chinese food chefs *N* = 330,947				Non-Chinese food chefs *N* = 47,179			
	Mean	SD	*N*	%	Mean	SD	*N*	%
Gender								
Female			231,270	69.9			27,245	57.8
Male			99,677	30.1			19,934	42.2
Age (years) at certification	32.19	11.79			23.27	9.38		
Age distribution								
15–39			232,134	70.1			43,211	91.6
40–59			97,339	29.4			3834	8.1
≥ 60			1474	0.5			134	0.3
Number of cancer diagnoses			5761				338	
Age at diagnosis (years)	48.09	9.28			42.37	11.79		
Person-years at risk^a^	11.08	2.71			11.54	4.54		

Lung cancer risk	Chinese food chefs *N* = 331,981				Non-Chinese food chefs *N* = 47,234			
	Mean	SD	*N*	%	Mean	SD	*N*	%
Gender								
Female			232,129	69.9			27,278	57.8
Male			99,852	30.1			19,956	42.2
Age (years) at certification	32.13	11.82			23.28	9.38		
Age distribution								
15–39			232,524	70.0			43,233	91.6
40–59			97,967	29.5			3865	8.2
≥ 60			1490	0.5			136	0.3
Number of cancer diagnoses			320				19	
Age at diagnosis (years)	51.99	8.45			44.73	12.57		
Person-years at risk^b^	11.53	4.54			11.07	2.72		

*SD* standard deviation

^a^Medians of person-years at risk were 11.07 years in Chinese food chefs and 10.42 years in non-Chinese food chefs

^b^Medians of person-years at risk were 11.07 years in Chinese-food chefs and 10.40 years in non-Chinese food chefs

Among the 4,183,550 and 4,220,163 person-years for the two risk groups, 6099 chefs developed cancer and 339 developed lung cancer (Tables 2, 3). The incidence rate (IR) of cancer was 158.3 per 100,000 person-years in Chinese food chefs and 62.3 per 100,000 person-years in non-Chinese food chefs (Table 2). Using the non-Chinese food chefs as a reference, we found Chinese food chefs to have a crude IRR of 2.54 (95% CI 2.28–2.84) for cancer. After adjusting for age and gender, the adjusted IRR was 1.69 (95% CI 1.51–1.89), which was significant (Table 2). The adjusted IRRs were 1.66 (95% CI 1.45–1.91) for females and 1.73 (95% CI 1.44–2.09) for males, both significant (Table 2).

**Table 2:** Incidence rate ratio (IRR) of cancer in total and by gender

	Person-years at risk (years)	Cancer					
		Number	IR/10^5^ person-year	Crude IRR	95% CI	Adjusted IRR^a^	95% CI
Total							
Non-Chinese food chefs	542,658	338	62.3	1.00		1.00	
Chinese food chefs	3,640,892	5761	158.3	**2.54**	**2.28–2.84**	**1.69**	**1.51–1.89**
Female							
Non-Chinese food chefs	310,107	216	69.7	1.00		1.00	
Chinese food chefs	2,542,241	4413	173.6	**2.49**	**2.17–2.86**	**1.66**	**1.45–1.91**
Male							
Non-Chinese food chefs	232,551	122	52.5	1.00		1.00	
Chinese food chefs	1,098,651	1349	122.8	**2.34**	**1.95–2.82**	**1.73**	**1.44–2.09**

Bold values indicate *p* < 0.05

*CI* confidence interval, *IR* incidence rate, *IRR* incidence rate ratio

^a^Adjusting for age range (15–39, 40–59 and ≥ 60 years old) and gender

The IR for lung cancer was 8.71 and 3.49 per 100,000 person-years in Chinese and non-Chinese food chefs, respectively (Table 3). Compared to non-Chinese food chefs, the crude IRR of Chinese food chefs was a significant 2.50 (95% CI 1.57–3.97). Although the adjusted IRR of lung cancer risk did not reach significance (1.46, 95% CI 0.91–2.34), risk was significantly higher among Chinese food chefs certified for > 5 years (adjusted IRR 2.12, 95% CI 1.32–3.40), compared to non-Chinese food chefs. IRR was significant for LA, but not for non-LA or LSCC solely (Table 3; eTable 3).

**Table 3:** Incidence risk ratio (IRR) of lung cancer and its subtypes in total and by gender

	Person-year at risk (years)	Lung cancer				LA				Non-LA^a^			
		Case	IR/10^5^ (years)	Crude IRR (95% CI)	Adjusted^b^ IRR (95% CI)	Case	IR/10^5^ (years)	Crude IRR (95% CI)	Adjusted^b^ IRR (95% CI)	Case	IR/10^5^ (years)	Crude IRR (95% CI)	Adjusted^b^ IRR (95% CI)
Total													
Non-Chinese food chefs	544,690	19	3.49	1.00	1.00	12	2.20	1.00	1.00	7	1.29	1.00	1.00
Chinese food chefs	3,675,473	320	8.71	**2.50 (1.57–3.97)**	1.46 (0.91–2.34)	223	6.07	**2.75 (1.54–4.92)**	1.26 (0.69–2.29)	97	2.64	2.05 (0.95–4.42)	1.37 (0.63–2.98)
Years of certification (years)													
≤ 5	1,656,756	65	3.92	1.12 (0.67–1.88)	0.66 (0.39–1.10)	44	2.66	1.21 (0.64–2.28)	**0.36 (0.19–0.70)**	21	1.27	0.99 (0.42–2.32)	0.66 (0.28–1.56)
					**2.12 (1.32–3.40)**								
> 5	2,018,717	255	12.63	**3.62 (2.27–5.77)**		179	8.87	**4.02 (2.24–7.22)**	**2.26 (1.24–4.10)**	76	3.76	**2.93 (1.35–6.35)**	1.95 (0.89–4.28)
Female													
Non-Chinese food chefs	311,439	4	1.28	1.00	1.00	3	0.96	1.00	1.00	1	0.32	1.00	1.00
Chinese food chefs	2,571,411	214	8.32	**6.48 (2.41–17.43)**	**3.32 (1.23–9.02)**	170	6.61	**6.86 (2.19–21.49)**	**3.56 (1.13–11.25)**	44	1.71	5.33 (0.73–38.68)	2.62 (0.35–19.42)
Years of certification (years)													
≤ 5	1,159,275	47	4.05	**3.16 (1.14–8.76)**	1.61 (0.58–4.51)	37	3.19	**3.31 (1.02–10.75)**	1.71 (0.52–5.60)	10	0.86	2.69 (0.34–20.99)	1.31 (0.17–10.47)
> 5	1,412,136	167	11.83	**9.21 (3.42–24.82)**	**4.73 (1.74–12.86)**	133	9.42	**9.78 (3.11–30.70)**	**5.08 (1.60–16.09)**	34	2.41	**7.50 (1.03–54.78)**	3.70 (0.50–27.57)
Male													
Non-Chinese food chefs	233,252	15	6.43	1.00	1.00	9	3.86	1.00	1.00	6	2.57	1.00	1.00
Chinese food chefs	1,104,062	106	9.60	1.49 (0.87–2.56)	0.95 (0.55–1.64)	53	4.80	1.24 (0.61–2.52)	0.81 (0.39–1.66)	53	4.80	1.87 (0.80–4.34)	1.15 (0.49–2.70)
Years of certification (years)													
≤ 5	497,481	18	3.62	0.56 (0.28–1.12)	**0.36 (0.18–0.72)**	7	1.41	**0.36 (0.14–0.98)**	**0.24 (0.09–0.65)**	11	2.21	0.86 (0.32–2.32)	0.53 (0.20–1.46)
> 5	606,581	88	14.51	**2.26 (1.30–3.90)**	1.42 (0.82–2.48)	46	7.58	1.97 (0.96–4.02)	1.27 (0.62–2.63)	42	6.92	**2.69 (1.14–6.33)**	1.65 (0.70–3.93)

Bold values indicate *p* < 0.05

*LA* lung adenocarcinoma

^a^Including small cell and non-small cell lung cancer except lung adenocarcinoma

^b^Adjusting for age range (15–39, 40–59, and ≥ 60 years old) and gender

Among the female chefs, Chinese food chefs certified for more than 5 years had a significant incidence of higher lung cancer, compared to non-Chinese food female chefs (adjusted IRR 4.73, 95% CI 1.74–12.86). The significance was particularly higher when analyzing the LA group of female workers (adjusted IRR 5.08, 95% CI 1.60–16.09). In contrast, these significant risks were not observed among male chefs regardless of type of cancer (Table 3; eTable 3).

## Discussion

This nationwide epidemiological study found that Chinese food chefs were at significantly higher risk of cancer than non-Chinese food chefs regardless of gender. Chinese food chefs who had received certificates for more than 5 years, especially female chefs, were also found to be at significantly increased risk of lung cancer.

COFs generated from high-temperature stir frying, pan frying and deep frying in oil not only contain known carcinogens and probable carcinogens but also inflammatory stimulators and/or reactive oxidative stress agents such as PAHs, volatile organic compounds (VOCs), aromatic amines, and long-chain aldehydes (IARC 2012; Yu et al. 2015). A few epidemiological studies have reported associations between COF exposure and cancer risk of other sites besides lungs, including the cervix and oral cavity in nonsmoking women (He et al. 2016; Lee et al. 2010; Wu et al. 2004; Xue et al. 2016). Because there is limited evidence of COF carcinogenicity in humans, IARC has categorized emissions from high-temperature frying as probably carcinogenic (IARC 2010). More evidence is needed. Our positive and significant findings of increased risk of cancer in Chinese food chefs, who are often exposed to long-term high concentrations of COFs, contributes importantly to the field’s evidence base regarding COF carcinogenicity in humans.

Chinese food chefs certified for more than 5 years had a significantly higher risk of lung cancer than non-Chinese food chefs, although significance was lost in those with few years (Table 2). The risk of LA was significantly higher in women with Chinese food chef certificates than those with non-Chinese food certificates (adjusted IRR 3.56; 95% CI 1.13–11.25), particularly so in those who had been certified more than 5 years (adjusted IRR 5.08; 95% CI 1.60–16.09), but not in those who had been certified ≤ 5 years (adjusted IRR 1.71; 95% CI 0.52–5.60). However, these significant differences were not found in males or in risk for non-LA or LSCC alone. The smoking rate is higher in men (21–39%) and lower in women (1.5–6.7%) in Taiwan (HPA 2014). Thus, we speculate that the effect of cigarette smoking on lung cancer risk may explain most of variability in males explaining the reason that COFs were associated with significant lung cancer risk in female Chinese chefs, not male chefs.

Most epidemiological studies have reported a positive association between exposure to COFs in home kitchens and the risk of lung cancers in nonsmoking women in Asian countries (Lee and Gany 2013; Mu et al. 2013; Sun et al. 2007). One earlier case–control study from Taiwan found that nonsmoking women who frequently cook at home were approximately two to fivefold more likely to have lung cancer than those who never cook at home (Ko et al. 2000). One study, basing their analysis on data collection by questionnaires, showed that nonsmoking women who serve as professional chefs have a similar lung cancer risk (~ two to fivefold), compared to those who were not professional chefs (Ko et al. 2000). Because COF risk can be affected by other covariates such as ventilation conditions and preferred cooking activities, etc., caution is needed with attempting to compare COF risk between home kitchens and commercial kitchens. In 2006, Yu et al. used frequency that one cooked, expressed in ‘dish-years’, as a proxy for cumulative COF exposure. In that study, they reported that, compared to nonsmoking Chinese women with ≤ 50 dish-years, those with > 200 dish-years had an odds ratio of 3.40 (95% CI 7.16–161.39) for developing lung cancer and 8.58 (95% CI 2.94–25.02) for developing LA (Yu et al. 2006). Subsequently, Lo et al. conducted a large-scale hospital-based case–control study in Taiwan with 1102 lung cancer patients and 1091 healthy controls (Lo et al. 2013), and found nonsmoking women who cooked more than 144 times per year to have a 1.78-fold (95% CI 1.14–2.78) higher risk of lung cancer than those who cooked less 20 times per year. They also found that installment of fume extractors at home kitchens protected against lung cancer risk in nonsmoking women (OR 0.57; 95% CI 0.35–0.94). In 2016, Xue et al. conducted a meta-analysis of data on 3596 nonsmoking female lung cancer patients and 6082 healthy nonsmoking women from 14 published case–control studies and found that exposure to COFs had the odds of 1.74 (95% CI 1.57–1.94) of developing lung cancer risk in nonsmoking women (Xue et al. 2016). That higher risk (OR 2.11; 95% CI 1.54–2.89) was also observed among women who cooked in kitchens without ventilators. The positive findings of this large-scale nationwide study of occupational chefs in workplaces provide another dimension of evidence on the effect of COFs on lung cancer risk.

The study has some limitations. One is that we did not have information on the smoking status of the participants identified in the database. This lack of information probably confounded the results more in male chefs than female ones, who have a relatively low rate of smoking in Taiwan. It is so low that the nationwide cancer study has even used sex as an appropriate surrogate variable of smoking status to examine the association of Chinese herbal products containing aristolochic acid and risk of urinary tract cancer (Lai et al. 2010). The current study is also limited in that we had no external comparison group free of COF exposure. Non-Chinese food chefs may possibly be exposed to COFs during work or while cooking at home. This could also occur in Chinese food chefs. These biases could possibly cause to underestimate the cancer risk of COFs. Another limitation is that although both Chinese and non-Chinese food chefs are required to adhere Taiwan’s labor law, we do not know how many hours they actually worked in the workplace. This could also confound the study results. One other limitation is lack of information about the time point that the chefs left work in both Chinese food and non-Chinese food chefs. These times were likely to be random and may result in some underestimation of COF cancer risk. Because this is a study of analysis secondary database, the causality of exposure and outcome of interest cannot be concluded. This study only examined total cancer and lung cancer risks in Chinese food chefs. Other cancer risks such as bladder cancer, colorectal cancer, and cervical cancer, etc., suggested by the previous studies, need to be explored in the future (Lee and Gany 2013; Wu et al. 2004). Still another limitation is that this single national study was conducted in a population of chefs with Han Chinese origin, caution is needed when attempting to generalize our findings to other populations.

In summary, this nationwide study of occupational chefs spanning more than 20 years found that the Chinese food chefs had higher total cancer risk than non-Chinese food chefs in both male and female. With regard to lung cancer, specifically, Chinese food chefs who had been certified for more than 5 years had a significantly higher risk than all non-Chinese food chefs. This risk was particularly higher in female Chinese food chefs. The highest LA risk was in female cooks who had been certified to cook Chinese food for more than 5 years. This is a study of analysis nationwide database, so it is necessary to conduct a prospective cohort study in occupational chefs to confirm the effect of COFs in lung cancer.

## Electronic supplementary material

Below is the link to the electronic supplementary material.

Supplementary material 1 (PDF 390 KB)
