# Supplementary material for: Gender differences and lung cancer risk in occupational chefs: analyzing more than 350,000 chefs in Taiwan, 1984–2011
Source: Int Arch Occup Environ Health. 2018 Sep 18;92(1):101–9. doi: 10.1007/s00420-018-1358-8 (PMC6323082; doi:10.1007/s00420-018-1358-8)
Supplement: Supplementary file 1 — Supplementary material 1 (PDF 390 KB) [file 420_2018_1358_MOESM1_ESM.pdf]

Tables

eTable 1. STROBE Statement—checklist of items regarding whether they were included in this observational study.

	Item No	Recommendation	Checklist
**Title and abstract**	1	(*a*) Indicate the study's design with a commonly used term in the title or the abstract	Yes
		(*b*) Provide in the abstract an informative and balanced summary of what was done and what was found	Yes
**Introduction**			
Background/rationale	2	Explain the scientific background and rationale for the investigation being reported	Yes
Objectives	3	State specific objectives, including any prespecified hypotheses	Yes
**Methods**			
Study design	4	Present key elements of study design early in the paper	Yes
Setting	5	Describe the setting, locations, and relevant dates, including periods of recruitment, exposure, follow-up, and data collection	Yes
Participants	6	(*a*) *Cohort study*—Give the eligibility criteria, and the sources and methods of selection of participants. Describe methods of follow-up<br>*Case-control study*—Give the eligibility criteria, and the sources and methods of case ascertainment and control selection. Give the rationale for the choice of cases and controls<br>*Cross-sectional study*—Give the eligibility criteria, and the sources and methods of selection of participants	Yes
		(*b*) *Cohort study*—For matched studies, give matching criteria and number of exposed and unexposed<br>*Case-control study*—For matched studies, give matching criteria and the number of controls per case	N/A
Variables	7	Clearly define all outcomes, exposures, predictors, potential confounders, and effect modifiers. Give diagnostic criteria, if applicable	Yes
Data sources/ measurement	8*	For each variable of interest, give sources of data and details of methods of assessment (measurement). Describe comparability of assessment methods if there is more than one group	Yes
Bias	9	Describe any efforts to address potential sources of bias	N/A
Study size	10	Explain how the study size was arrived at	Yes
Quantitative variables	11	Explain how quantitative variables were handled in the analyses. If applicable, describe which groupings were chosen and why	Yes
Statistical methods	12	(*a*) Describe all statistical methods, including those used to control for confounding	Yes
		(*b*) Describe any methods used to examine subgroups and interactions	Yes
		(*c*) Explain how missing data were addressed	N/A
		(*d*) *Cohort study*—If applicable, explain how loss to follow-up was addressed<br>*Case-control study*—If applicable, explain how matching of cases and controls was addressed<br>*Cross-sectional study*—If applicable, describe analytical methods taking account of sampling strategy	N/A
		(*e*) Describe any sensitivity analyses	N/A
**Results**			
Participants	13*	(a) Report numbers of individuals at each stage of study—eg numbers potentially eligible, examined for eligibility, confirmed eligible, included in the study, completing follow-up, and analysed	Yes
		(b) Give reasons for non-participation at each stage	Yes
		(c) Consider use of a flow diagram	Yes
Descriptive data	14*	(a) Give characteristics of study participants (eg demographic, clinical, social) and information on exposures and potential confounders	Yes
		(b) Indicate number of participants with missing data for each variable of interest	Yes
		(c) *Cohort study*—Summarise follow-up time (eg, average and total amount)	Yes
Outcome data	15*	*Cohort study*—Report numbers of outcome events or summary measures over time	Yes
		Case-control study—Report numbers in each exposure category, or summary measures of exposure	N/A
		Cross-sectional study—Report numbers of outcome events or summary measures	N/A

Main results	16	(*a*) Give unadjusted estimates and, if applicable, confounder-adjusted estimates and their precision (eg, 95% confidence interval). Make clear which confounders were adjusted for and why they were included	Yes
		(*b*) Report category boundaries when continuous variables were categorized	Yes
		(*c*) If relevant, consider translating estimates of relative risk into absolute risk for a meaningful time period	Yes
Other analyses	17	Report other analyses done—eg analyses of subgroups and interactions, and sensitivity analyses	Yes
Discussion			
Key results	18	Summarise key results with reference to study objectives	Yes
Limitations	19	Discuss limitations of the study, taking into account sources of potential bias or imprecision. Discuss both direction and magnitude of any potential bias	Yes
Interpretation	20	Give a cautious overall interpretation of results considering objectives, limitations, multiplicity of analyses, results from similar studies, and other relevant evidence	Yes
Generalisability	21	Discuss the generalisability (external validity) of the study results	N/A
**Other information**			
Funding	22	Give the source of funding and the role of the funders for the present study and, if applicable, for the original study on which the present article is based	Yes

eTable 2. Lung cancer categorized by histological subtypes (ICD-9-CM: 162).

Histological Type	Group code[1]	Histological type
Small cell carcinoma	1	8041
Combined cell carcinoma	1	8045
Adenocarcinoma	2	8140
Bronchioloalveolar carcinoma	2	8250
Adenoid alveolar adenocarcinoma	2	8251
Adenocarcinoma, mixed subtype	2	8255
Papillary adenocarcinoma	2	8260
Mucinous (“colloid”) carcinoma	2	8480
Squamous cell carcinoma	3	8070
Keratinzing squamous cell carcinoma	3	8071
Nonkeratinzing squamous cell carcinoma	3	8072
Large cell carcinoma	4	8012
Pleomorphic carcinoma	4	8022
Sarcomatoid carcinoma	4	8033
Bronchioloalveolar nonmucinous carcinoma	4	8252
Mucoepidermoid carcinoma	4	8430
Acinar adenocarcinoma	4	8550
Adenosquamous carcinoma	4	8560
Spindle cell sarcom	4	8801
Pulmonary blastoma	4	8972
Marginal zone B-cell lymphoma of the MALT type	4	9699
Carcinoma	4	8000
Unspecified malignant neoplasm	4	8010

[1]Group code: 1 indicates small cell; 2 indicates non-small cell and adenocarcinoma; 3 indicates non-small cell and squamous cell carcinoma; 4 indicates non-small cell and others.

References from https://www.iarc.fr/en/publications/pdfs-online/epi/sp160/CI5vol9-4.pdf and https://www.iarc.fr/en/publications/pdfs-online/pat-gen/bb10/bb10-chap1.pdf

eTable 3. Incidence risk ratio (IRR) of lung squamous cell carcinoma (LSCC) in total and by gender.

		Person year at risk (yrs)	LSCC			
			Case	IR/10^5 (yrs)	Crude IRR (95% CI)	Adjusted[1] IRR (95% CI)
**Total**						
**Non-Chinese food chefs**		544,690	5	0.92	1.00	1.00
**Chinese food chefs**		3,675,473	28	0.76	0.83 (0.32-2.15)	0.55 (0.21-1.46)
**Years of certification (yrs)**	≤ 5	1,656,756	2	0.12	**0.13 (0.03-0.68)**	**0.09 (0.02-0.46)**
	> 5	2,018,717	26	1.29	1.40 (0.54-3.65)	0.92 (0.34-2.48)
**Female**						
**Non-Chinese food chefs**		311,439	1	0.32	1.00	1.00
**Chinese food chefs**		2,571,411	10	0.39	1.21 (0.16-9.46)	0.41 (0.05-3.40)
**Years of certification (yrs)**	≤ 5	1,159,275	1	0.09	0.27 (0.02-4.30)	0.09 (0.01-1.51)
	> 5	1,412,136	9	0.64	1.99 (0.25-15.67)	0.67 (0.08-5.66)
**Male**						
**Non-Chinese food chefs**		233,252	4	1.71	1.00	1.00
**Chinese food chefs**		1,104,062	18	1.63	0.95 (0.32-2.81)	0.59 (0.19-1.77)
**Years of certification (yrs)**	≤ 5	497,481	1	0.20	0.12 (0.01-1.05)	**0.07 (0.01-0.66)**
	> 5	606,581	17	2.80	1.63 (0.55-4.86)	1.00 (0.33-3.05)

[1]Adjusting for age range (15-39, 40-59 and ≥ 60 years old) and gender.
